# Repeated Successful Nest Sharing and Cooperation Between Western Kingbirds (*Tyrannus verticalis*) and a Female Western Kingbird × Scissor‐Tailed Flycatcher (*T. forficatus*) Hybrid

**DOI:** 10.1002/ece3.70818

**Published:** 2025-01-14

**Authors:** Alexander J. Worm, Emily R. Donahue, Than J. Boves, Andrew D. Sweet

**Affiliations:** ^1^ Department of Biological Sciences Arkansas State University Jonesboro Arkansas USA

**Keywords:** avian reproduction, cooperative breeding, hybridization, paternity analysis, SNPs, whole‐genome

## Abstract

Nest sharing by birds, or the phenomenon where multiple individuals of different species contribute genetically and parentally to offspring in a single nest, is a rare form of cooperative breeding that has only occasionally been reported in socially monogamous birds. Here we describe, both behaviorally and genetically, the unique case of two female birds, a western kingbird ( *Tyrannus verticalis* ) and a western kingbird × scissor‐tailed flycatcher ( *T. forficatus* ) hybrid, simultaneously occupying (and likely co‐incubating eggs in) a single nest. Both females provisioned nestlings, and they did this in two consecutive years (producing four fledglings each year). Genomic data from the females revealed that they were unrelated, and parentage analyses revealed that both females contributed genetically to at least one of the offspring, and at least two fathers were involved. These observations represent the first reported case of nest sharing involving a hybrid individual and the first case within the family Tyrannidae.

## Graphical abstract

This note describes, both behaviorally and genetically, a unique case of two female birds, a western kingbird ( *Tyrannus verticalis* ) and a western kingbird × scissor‐tailed flycatcher ( *T. forficatus* ) hybrid, simultaneously occupying (and likely co‐incubating) a brood in a single nest across multiple breeding seasons. Genomic analyses revealed that both females and two offspring sampled were unrelated, and the offspring were sired by different males. This is the first report of cooperative breeding involving a hybrid individual.

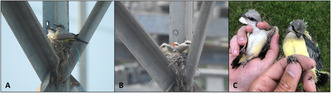

## 1 Introduction

The reproductive behaviors of birds are incredibly diverse and have been generally well‐documented across a large percentage of species on Earth (Kempenaers 2022). Although most bird species are socially monogamous (> 80%; Cockburn 2006), considerable variation exists (Owens and Bennett 1997; Kempenaers 2022). Furthermore, atypical reproductive behaviors likely remain under‐reported, resulting in an incomplete view of avian mating systems as a whole. Cooperative breeding, where two or more adults contribute parentally to a single nest, is one example of an atypical mating system in birds (Koenig et al. 1992; Vehrencamp 2000). Cooperative breeding has been documented in about 9% of bird species (Cockburn 2006). Two types of cooperative breeding systems are joint‐nesting and nest sharing. Joint‐nesting is an evolutionarily stable breeding strategy that involves multiple individuals of the same sex and species sharing a single nest. In species known to participate in this behavior, joint‐nesting is typically the primary breeding strategy used by those species (Vehrencamp 2000). Well‐documented examples of joint‐nesting in birds include anis (*Crotophaga* spp.; Riehl and Jara 2009), Seychelles warblers ( *Acrocephalus sechellensis* ; Van Boheemen et al. 2019), and acorn woodpeckers ( *Melanerpes formicivorus* ; Vehrencamp 2000). A much rarer form of cooperative breeding is nest sharing, which is when two or more bird species contribute some parental care to offspring in a single nest (Mulvihill and Murray 2022). Many examples of nest sharing involve cavity‐nesting species and are attributed to competition for limited nest sites (Samplonius and Both 2014; Barrientos, Bueno‐Enciso, and Sanz 2015). Examples of nest sharing involving open‐cup nesting passerines also exist, but these cases typically involve species that are from different genera (Raney 1939; Brackbill 1952; Govoni, Summerville, and Eaton 2009; Mulvihill and Murray 2022). Only two cases of intra‐generic nest sharing involving open‐cup nesting passerines have been previously documented (*Turdus*; Freemann and Batten 1970, *Phoenicurus*; Bruni, Mori, and Balestrieri 2023).

Although descriptions of nest sharing in birds exist in the literature, understanding the causes and consequences of these behaviors has been challenging for several reasons. First, nest sharing events are only rarely observed and even less often monitored to document parental nesting behaviors and determine nest fate. Second, most accounts of nest sharing describe observations without providing information regarding the heredity of adults or offspring. Given the rarity of nest sharing, fitness costs of this behavior are typically assumed to be high for the individuals involved, however, relatedness among individuals could balance out high individual fitness costs (i.e., kin selection; Koenig et al. 2023). Determining potential costs to test this scenario requires knowledge of the relatedness among involved individuals which is often unknown in documented cases. Costs could include reduced fitness via reduced reproductive output and via potential injury (e.g., confrontations with territory holders/established nesters; Koenig et al. 1995). However, it is possible that nest sharing could benefit some individuals that are unable to secure their own mates or nesting locations and that would have otherwise foregone a reproduction attempt altogether (Gowaty 1981). Additionally, nest sharing could be beneficial when nesting success is greater in joint nests due to cooperative nest defense (Riehl 2010). However, the specific costs and benefits of nest sharing are likely context‐dependent, and the lack of observations of the same individuals tracked over multiple breeding seasons makes it difficult to test specific hypotheses.

One context where nest sharing could be beneficial is in cases involving hybrid individuals. Hybrids, defined here as offspring resulting from the interbreeding of two distinct species, or subsequent backcrossing, often suffer from reduced reproductive output that may be caused, in part, by the inability to secure a mate (Rhymer and Simberloff 1996; Price and Bouvier 2002; Svedin et al. 2008). Therefore, hybrid individuals may be ideal candidates to gain from nest sharing; in this case, categorizing nest sharing as intra‐ or inter‐specific is challenging because hybrids fall in the middle of this dichotomy. However, although hybridization is known to occur in 15%–20% of avian species (Ottenburghs 2023), there are currently no documented cases of nest sharing involving a hybrid individual. This may be because detecting hybrid individuals often requires genetic information, which can be challenging to obtain especially when hybrid individuals are rare.

Here, we describe the first reported case of nest sharing involving a hybrid individual and the first case involving a passerine species in the most diverse family of birds, *Tyrannidae*. Specifically, we describe observations associated with repeated nest sharing by a female hybrid western kingbird ( *Tyrannus verticalis* ) × scissor‐tailed flycatcher ( *T. forficatus* ). Further, we also obtained and analyzed genomic information to characterize the relationships between the two females and offspring and followed these individuals for multiple breeding seasons to evaluate reproductive success. With this data, this account is among the most thorough documentations of nest sharing.

## 2 Materials and Methods

### 2.1 Observation and Data Collection

**FIGURE 1:**
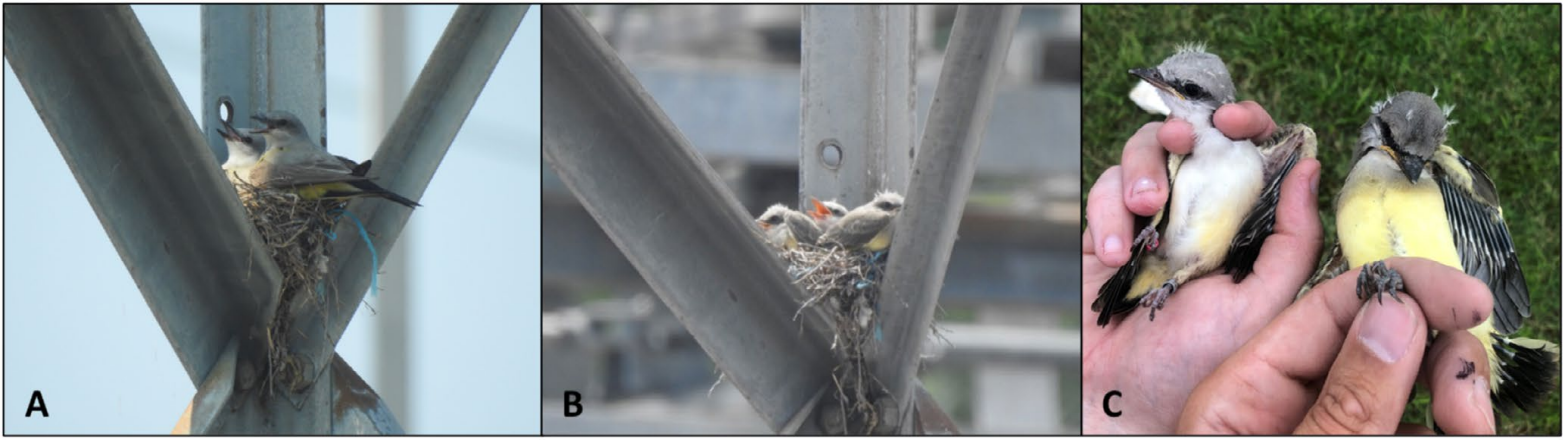
(A) Female scissor‐tailed flycatcher ( *Tyrannus forficatus* ) × western kingbird ( *T. verticalis* ; left) hybrid co‐incubating a shared nest with a female western kingbird (right) in Bald Knob, Arkansas; (B) brood of nestlings resulting from the nest‐sharing attempt; (C) two of the fledglings, Fledgling A (left; admixed) and Fledgling B (right; pure western kingbird), from the joint‐nest brood that was used in the parentage analysis.

Scissor‐tailed Flycatchers and Western Kingbirds are Nearctic‐Neotropical migrant sister species (Harvey et al. 2020) that are largely sympatric, breeding throughout the central United States in open and semi‐open habitats (Regosin and Pruett‐Jones 1995; Gamble and Bergin 2020). Both species are socially monogamous with no reports of polygyny, but each species experiences high rates of extra‐pair paternity (Roeder, Husak, and Murphy 2016). Both species are simultaneously expanding their ranges eastward, and hybridization between the two species has been ongoing at the eastern peripheries of both species' ranges since at least 2004 (Sullivan et al. 2009; Worm et al. 2019). We have been monitoring birds in this “hybrid zone” in some capacity since 2014. On 13 June 2021, we observed two female birds simultaneously sitting on a nest within an electrical substation located in Bald Knob, Arkansas, USA (35.32844, −91.56174; Figure 1). Both females had previously been captured and uniquely marked with United States Geological Survey (USGS) aluminum and plastic‐colored bands in 2016, so we were certain of their identities (Worm et al. 2019). One of the females was a (genetically confirmed via microsatellites and mtDNA; Worm et al. 2019) western kingbird × scissor‐tailed flycatcher hybrid (hereinafter referred to as “hybrid female”) that had been nesting at the exact location annually since 2015; she was ≥ 8 years old as of 2021. Over multiple breeding seasons, the hybrid female successfully fledged several nests, pairing with putative pure male scissor‐tailed flycatchers in some years and putative pure male western kingbirds in others (AJW pers. obvs.). The second female was a pure (also genetically confirmed with microsatellites and mtDNA; Worm et al. 2019) western kingbird (hereinafter “pure female”) that was captured and banded as a fledgling in 2016 from a nest ~2.6 km away (35.34306, −91.53915); she was 6 years old as of 2021. The shared nest was located ~4.5 m above the ground in the cross arms of a steel substation structure (Figure 1A), and although we cannot be certain, both females appeared to be incubating on this date; both were sitting low in the nest for over an hour, with the hybrid female sitting squarely on the back of the pure female. At the time of this observation, our hypothesis was that the two females were engaged in a dispute over ownership of the nest or social mate (a putative pure western kingbird), and one would eventually yield to the other and abandon her attempt. However, over the next 10 days, both the pure and hybrid females continued to co‐occupy the nest (again, likely co‐incubating based on our observations) without demonstrating any agonistic behavior toward each other for the entirety of the nesting attempt.

On 24 June 2021, we observed nestlings (four) while both females simultaneously brooded (Figure 1B) with one female on the young and the second female sitting on the back of the other. Both females, and what we believed to be a single, unbanded social male western kingbird, fed the young indiscriminately and approximately equally over the next 15 days, which represents a typical nestling period length for both species (Regosin and Pruett‐Jones 1995; Gamble and Bergin 2020). On 9 July 2021, four young fledged from the nest; we then captured all four fledglings and extracted ~100 μL of blood from the brachial vein, which we stored in a lysis buffer for later molecular analysis. We gave each fledgling a uniquely numbered USGS aluminum band and a 2‐color band combination for potential future identification of these individuals in the field. Based on variation in the amount of yellow and gray plumage of the fledglings, we morphologically identified three as putative pure western kingbirds and one as an admixed hybrid (Figure 1C).

During the following breeding season, we returned to the site on 1 May 2022 and relocated both females (but none of the four banded offspring from 2021). On 4 June 2022, we observed both females again co‐occupying the same nest location, the same that they had used in the previous season, with an unbanded western kingbird social male. We monitored this nest for the next month as well; both females behaved similarly to the previous year (with respect to incubation, brooding, and provisioning), and once again, four young fledged. However, we were unable to capture the fledglings this season. We returned to the site again in 2023 and observed the pure female nesting in the same substation but in a different nest location; we were unable to locate the hybrid female or any of the banded offspring from 2021.

Notably, the hybrid female was ≥ 9 years old when we last observed her in 2022. This would be older than the longevity records for either parental species. For western kingbirds, the current documented longevity record is ≥ 6 years and 11 months old (Gamble and Bergin 2020), which was also surpassed by the pure female from this study at 8 years old in 2023; for scissor‐tailed flycatchers, the current record is ≥ 6 years old (Becker et al. 2018).

### 2.2 Genomic Analysis: Sample Collection and Processing, Genome‐Wide Variant Calling, and Parentage Analysis

To assess whether each female contributed genetically to the offspring and whether the offspring shared the same father, we used blood samples collected both during the current study and during prior studies. We analyzed blood samples from two of the four offspring from the joint nest: the one that we morphologically identified as admixed (hereinafter fledgling A) and one of the three putative “pure” offspring (hereinafter fledgling B; Figure 1C). For the adult females, we analyzed blood samples collected during a previous study (Worm et al. 2019). Since small sample sizes of closely related individuals can lead to biased relatedness analyses, we also included four western kingbird tissue samples that were collected from various parts of their range to increase the accuracy of the relatedness analysis (Wang 2017). Of the four additional samples, was a blood sample we collected from an adult in Memphis, TN, and the other three were muscle samples from the Louisiana State University Museum of Natural Science (LSUMNS) that were collected outside of the hybrid zone in California (LSUMNS 53166, 53203, and 53194).

From these eight samples, we generated genome‐wide single nucleotide polymorphism (SNP) data. We extracted genomic DNA using a DNEasy Blood and Tissue Kit (Qiagen, Hilden, Germany) following the manufacturer's protocol. We then assessed the quantity of extracted DNA using a fluorometric assay (DeNovix, Wilmington, Delaware). Genomic DNA was sent to Novogene (Sacramento, California) for library preparation and sequencing. Indexed samples were pooled and sequenced on an Illumina NovaSeq 6000 S4 platform, generating 150 bp paired‐end reads with a target coverage of 5×. We trimmed sequenced reads using Trimmomatic version 0.39 (Bolger, Lohse, and Usadel 2014), with leading:3, trailing:3, slidingwindow:4:15, and minlen:75, and removed duplicate reads using the dedupe2 script distributed with BBMap version 38.82 (Bushnell 2014). We checked the quality of reads using FastQC v0.11.5 (Babraham Bioinformatics) before and after trimming. We then mapped the trimmed reads to a high‐coverage scissor‐tailed flycatcher genome assembly (GenBank: PRJNA802243) using the “end‐to‐end” parameter in Bowtie2 version 2.4.1 (Langmead and Salzberg 2012). We converted individual SAM files into BAM files using SAMtools version 1.15.1 (Danecek et al. 2021) and jointly called filtered SNP datasets in BCFtools version 1.10.2 (Danecek et al. 2021). SNPs were then filtered in BCFtools and VCFtools version 0.1.15 (Danecek et al. 2011) based on depth < 20 & > 100, quality < 28, mapping quality < 20, missing data, and keeping only biallelic SNPs, and we removed SNPs if they were not shared by at least one other individual.

We performed two parentage analyses to determine relatedness among the two adult females and the two sampled fledglings. For the first parentage analysis, we used the filtered SNP dataset to perform a kinship coefficient analysis between all pairs using *relatedness2* in VCFtools to estimate the KING relatedness coefficient. The KING coefficient is commonly used to determine degrees of relatedness between individuals (Manichaikul et al. 2010). A coefficient > 0.354 indicates self or twins, 0.177–0.354 indicates first‐degree relatedness (i.e., offspring, parents, full siblings), 0.0884–0.177 indicates second‐degree relatedness (i.e., grandparents, aunts/uncles, grandchildren, niece/nephew, half‐siblings), 0.0625 indicates third‐degree relatedness (e.g., first cousin), and negative values indicate no genetic relatedness to fifth‐degree relatedness. For the second parentage analysis, we determined maternity for the fledglings by generating a phylogeny and uncorrected p‐distances for both adult females and the two fledglings incorporating the 13 mitochondrial protein genes. The uncorrected p‐distance measures the raw genetic distance between two individuals via the number of nucleotide differences in the mtDNA protein‐coding genes. To generate the p‐distance measures, we assembled the mitochondrial genomes from both adult females and the two fledglings using MITObim, annotated each assembly in MITOS (Bernt et al. 2013), and completed the same mitogenomic analysis for a blood sample from a Scissor‐tailed Flycatcher captured in Lawton, Oklahoma during the 2022 breeding season as an outgroup. We used a scissor‐tailed flycatcher ND2 gene as the seed for MITObim (GenBank: MH747741.1). We visualized the mitogenomes in Geneious Prime version 2022.0.2 to confirm annotations of the protein‐coding genes based on open reading frames. We then aligned each gene using default parameters in MUSCLE version 5.1 (Edgar 2004), concatenated the genes in Geneious Prime, calculated uncorrected p‐distances, and constructed a neighbor‐joining distance tree using the R package ape version 5.7.1 (R Core Team 2023) with the K80, TN93, and JC substitution models.

## 3 Results

**FIGURE 2:**
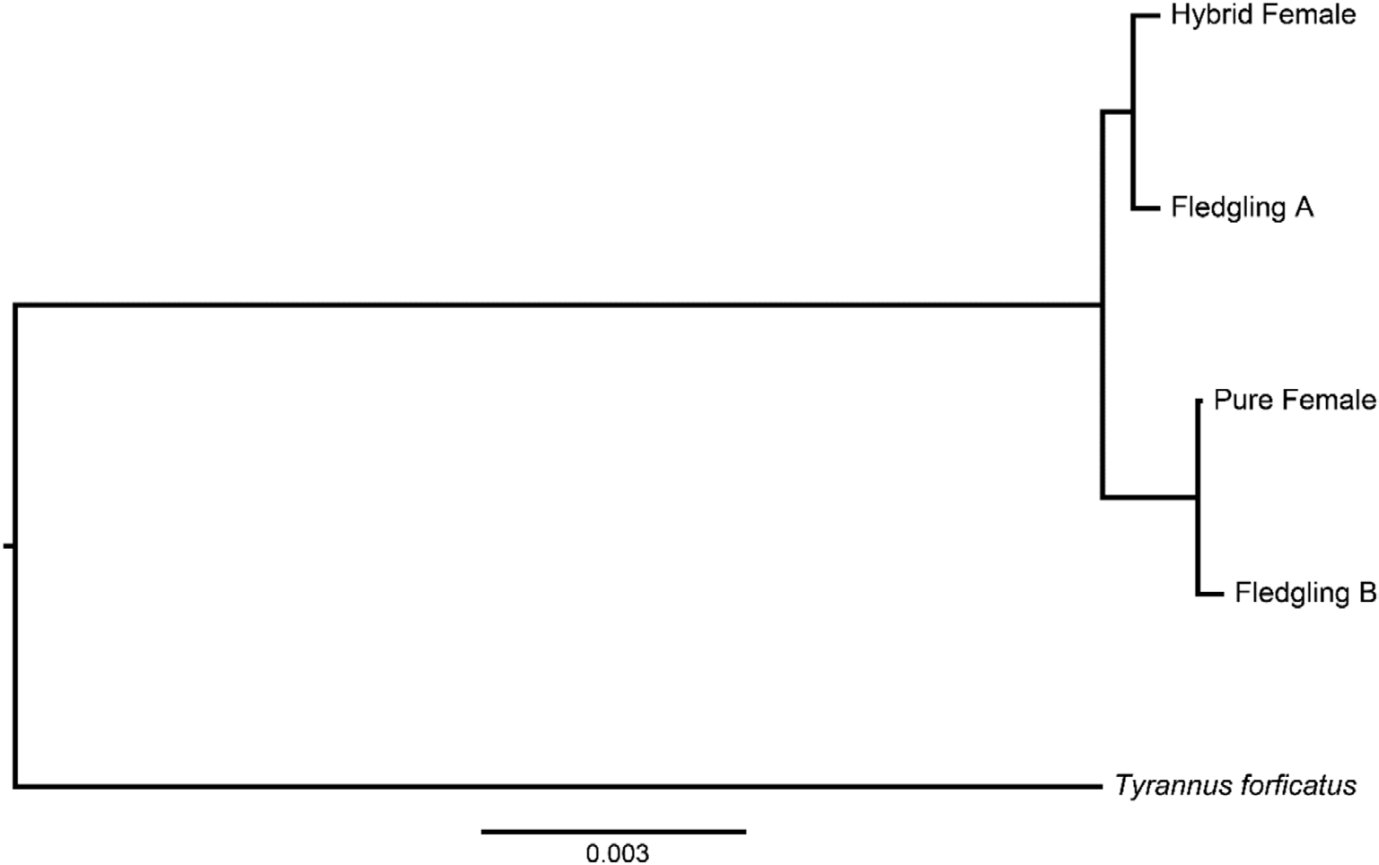
A neighbor‐joining distance tree from 13 mitochondrial protein genes of a nest‐sharing pure female western kingbird ( *Tyrannus verticalis* ; pure female) and scissor‐tailed flycatcher ( *T. forficatus* ) × western kingbird hybrid (hybrid female), and two offspring from the shared nest (Fledgling A and B). The tree is rooted on a scissor‐tailed flycatcher.

Our genome‐wide SNP data for the eight individuals comprised 14,838,408 SNPs and 8,371,835 SNPs before and after filtering, respectively, with an average sequencing depth of 7× and mapping coverage of > 96% in all samples. Based on our parentage analysis using the pairwise kinship coefficients, we determined that fledgling A was an offspring of the hybrid female and fledgling B was an offspring of the pure female (Table 1; first degree of relatedness coefficients both > 0.177). Our mitochondrial data further supported these relationships, with fledgling A grouping with the hybrid female (p‐distance 0.0006) and fledgling B grouping with the pure female (p‐distance 0.0004, Figure 2, Table 2). The uncorrected p‐distance between the two adults was 0.002; the distance between the two fledglings was also 0.002 (Table 2). Furthermore, the same topology was consistently recovered across all three substitution models used for the neighbor‐joining distance tree. These results from the nuclear and mitochondrial sequence data support our initial assessment that one fledgling (fledgling A) had admixed ancestry whereas the other (fledgling B) was a pure western kingbird. Additionally, our parentage analyses supported that both fledgling A and B were unrelated, and the two adult females were unrelated, with both pairwise comparisons being negative relatedness coefficients (zero or negative coefficient indicating more than fifth‐degree relatedness; Table 1). The fledglings were neither full nor half‐siblings, indicating that the offspring in the shared nest were sired by at least two males (Table 1). Although we did not sequence DNA from the other two fledglings, they were morphologically more similar in appearance to fledgling B (offspring of the pure female) based on the level of yellow and gray plumage coloration differences (Figure 1C). Therefore, the pure female likely produced three offspring, and the hybrid female likely produced one offspring in their shared nest.

**TABLE 1:** Kinship coefficients of two adult females and two offspring from a shared nest in Arkansas.

	Pure female	Hybrid female	Fledgling A	Fledgling B
Pure female	0.5			
Hybrid female	−0.1421	0.5		
Fledgling A	−0.5017	**0.3228**	0.5	
Fledgling B	**0.1856**	−0.1205	−0.4676	0.5

*Note:* Pure female is a western kingbird ( *Tyrannus verticalis* ), hybrid female is a hybrid scissor‐tailed flycatcher ( *T. forficatus* ) × western kingbird, and Fledgling A and B are two of the four offspring from the nest. Values indicating second‐degree relatedness (parent–offspring) are in bold. A negative coefficient indicates more than fifth‐degree relatedness.

**TABLE 2:** Uncorrected p‐distances of two adult females and two offspring from a shared nest in Arkansas using the 13 mitochondrial protein‐coding genes.

	Pure female	Hybrid female	Fledgling A	Fledgling B
Pure female				
Hybrid female	0.00187			
Fledgling A	0.00178	0.00062		
Fledgling B	0.00037	0.00205	0.00196	

*Note:* Pure female is a western kingbird ( *Tyrannus verticalis* ); hybrid female is a hybrid scissor‐tailed flycatcher ( *Tyrannus forficatus* ) × western kingbird; and Fledgling A and B are two of the four offspring from the nest. Larger values indicate greater genetic differences (more distantly related), while smaller values indicate smaller genetic differences (more closely related).

## 4 Discussion

Here, we thoroughly documented and described the first case of a hybrid bird and the first case of a member of Tyrannidae participating in nest sharing. Our observation is also one of a few accounts of nest sharing attempts that involved open‐cup nesting, passerine birds from two taxa within the same genus (Freemann and Batten 1970; Bruni, Mori, and Balestrieri 2023). However, this account represents a unique case because one individual was a hybrid. In addition to being incredibly rare, descriptions of nest sharing are typically limited in their ability to infer hereditary information about the adults and offspring involved because of a lack of associated molecular data. In our study, incorporating molecular data allowed us to determine the degree of relatedness among the parents and offspring known to have shared the nest, which can help explain (or eliminate) potential causes of the behavior (Theuerkauf et al. 2009).

Several hypotheses have been proposed to explain the evolution of various cooperative breeding systems in birds. In joint‐nesting species like the acorn woodpecker, cooperative breeding can typically be adaptively explained by kin selection (and inclusive fitness; Koenig et al. 2023) and ecological constraints (e.g., nest site and/or food availability; Barve et al. 2019). Additionally, greater anis ( *Crotophaga major* ) seem to cooperatively breed to increase nest defense, as communal nests have greater success compared to those of single pairs (Riehl 2010). In our study, kin selection does not seem to apply, as the two females were unrelated (more than fifth‐degree relatedness), and the two sampled offspring were also unrelated (Table 1). This indicates that the offspring were sired by unrelated males, although we cannot rule out the possibility that the social male was related to one of the females and "allowed" her to share the nest (Clutton‐Brock 2002; Hatchwell 2009). Additionally, Ecological constraint does not seem to be occurring in our system, as there were numerous nest sites available to each of the females (author per. obs.). Moreover, enhanced nest defense is likely not the main explaination for nest sharing in this case, although it cannot be entirely ruled out. Western kingbirds in our study system were previously documented to have relatively high nest success (Worm et al. 2019). Finally, when examining previous cases of nest sharing in open‐cup passerines, such as between a northern cardinal ( *Cardinalis cardinalis* ) and a song sparrow ( *Melospiza melodia* ; Brackbill 1952), and a gray catbird ( *Dumetella carolinensis* ) and an American robin ( *Turdus migratorius* ; Mulvihill and Murray 2022), the most common explanation for this behavior is that one of the females of the pair involved mistakenly laid eggs into the wrong nest (Lindell 1996). The fact that we observed the same two females participated in nest sharing in two breeding seasons likely rules out mistakenly laying eggs as a reason for at least the second attempt.

Although speculative, several non‐exclusive circumstances could have led to the nest‐sharing cases we observed, none of which seem to involve adaptive behavior by all parties involved. One female (likely the hybrid) may have had difficulty obtaining a social mate, so she made the “best of a bad situation” by sharing the social male, and subsequently, the nest, of a nearby female. This scenario seems most likely based on our experience with the *T. verticalis* × *T. forficatus* system. Over the past decade of monitoring these species across the hybrid zone (eastern Arkansas and western Tennessee), we have observed multiple hybrid females often struggle to secure a social mate and resort to repeatedly nesting close to a pure social western kingbird pair (presumably sharing the social male, author pers. obs). Furthermore, low densities of available mates have been observed across our study system, which is the eastern periphery of the western kingbird's range (author pers. obs.; Sullivan et al. 2009). Thus, it seems a female unable to secure a social mate would benefit from not foregoing a breeding season (Gowaty 1981). Additional non‐exclusive explanations include an initial failed nesting attempt by one of the females (Wiens 1971) or one of the females laying eggs in an occupied nest without the knowledge of the other female (Sealy and Bazin 1995). However, these explanations fail to adequately explain why the same females shared a nest at the same site across multiple breeding seasons.

The case of nest sharing described here is an important contribution to better understanding the variation of nesting behaviors in birds. By combining observations of known individuals over multiple years with information on genetic relatedness, we expand on known breeding strategies in birds and shed light on some potential evolutionary explanations for shared nesting behavior. However, to further broaden our understanding of nest sharing in birds, more documentation is needed on cases of nest sharing, including those involving hybrid individuals and those spanning multiple breeding seasons. Additionally, more field observations paired with genetic analyses in cases of nest sharing will help clarify the evolutionary causes and consequences of cooperative breeding tactics in birds.

## Author Contributions

**Alexander J. Worm:** conceptualization (equal), data curation (lead), formal analysis (lead), funding acquisition (lead), investigation (equal), methodology (lead), project administration (lead), software (lead), writing – original draft (equal), writing – review and editing (lead). **Emily R. Donahue:** conceptualization (equal), investigation (equal), methodology (equal), writing – original draft (equal), writing – review and editing (equal). **Than J. Boves:** conceptualization (equal), funding acquisition (supporting), writing – review and editing (supporting). **Andrew D. Sweet:** conceptualization (equal), data curation (supporting), formal analysis (supporting), software (supporting), supervision (supporting), writing – review and editing (supporting).

## Conflicts of Interest

The authors declare no conflicts of interest.

## Data Availability

Raw genomic data for all nine samples is publicly available via NCBI Sequence Read Archive under the BioProject ID PRJNA1141522 (SRR30213066–SRR30213074). All scripts and input files needed to recreate the analyses used in this manuscript are stored in Dryad: https://doi.org/10.5061/dryad.w6m905qz.
